# Preventive and Rescue Work

**Published:** 1922-07

**Authors:** 


### PREVENTIVE AND RESCUE WORK.

### A FORWARD MOVE IN LONDON.

A NOTABLE step was taken recently in the setting up of a "Central Council on Rescue and Preventive Work in London." On this Council representatives of the Ministry of Health, the Home Office, the London County Council, the City Corporation, the Metropolitan Asylums Board, and other public authorities will meet representatives of the principal voluntary bodies engaged in rescue and preventive work, to consider questions in which they have a common interest, and to evolve that concerted action without which no real progress is possible.

A few years ago it would have been argued that Government Departments and Local Authorities had no interest in the work of voluntary rescue societies, and the change of outlook which has led to the formation of the Central Council is itself significant. To-day it is recognised that the effects of sexual immorality constitute a grave social problem which must be met by many-sided action, and that social, moral and spiritual forces must all be harnessed together for this task.

The decision to form the Central Council was taken at a conference called by the Minister of Health, and held at the offices of the Ministry, but a good deal of preliminary work had already been done before that point was reached. A year or more ago a Provisional Committee on Rescue and Preventive Work was formed at a conference convened by the National Council of Social Service. Later, this Committee was received in deputation by the Public Health Committee of the London County Council, and submitted proposals for the establishment of a permanent representative Council to secure closer co-operation between public and voluntary bodies. Following this the London County Council resolved to favour the suggestion that a central joint committee should be formed representative of public authorities, and of voluntary agencies concerned in rescue and preventive work in London. From this step negotiations with the Ministry of Health were conducted jointly by representatives of the County Council and of the Provisional Committee, with the result already recorded. Lord Astor is President of the new Council, and Captain O. S. Warburg (Chairman of the Public Health Committee of the London County Council) is Chairman.

The Council is in the early stage of development, and has indeed hardly begun work. It has been decided, wisely, that its first step must be to prepare a preliminary review of the problem, its nature and extent, and of the existing provision to meet it. Only when this has been done will it be possible to compare provision and need, to see how far the former is adequate or where it is deficient, and to formulate a more complete scheme in which all the forces available will be used to their fullest advantage. The problem is one in which the health authorities are intimately concerned because of the relation between sexual immorality and venereal disease.

The problem is also closely related to others such as illegitimacy and mental deficiency, which come directly under its purview. Thus, in the notifications to the London County Council under the Mental Deficiency Act, 1913, there was "clear evidence of immorality in 265 cases."* As illustrating the variety of agencies interested in this particular group of women and girls, it is significant that the sources of notification were as follows: Prison, 3; Police Court, 57; Poor Law, 81; Voluntary Refuges, 74; Miscellaneous, 50.

It would be unwise to attempt to anticipate the comprehensive scheme which the Central Council has set out to evolve, but there seem to be certain provisions required by the very nature of the problem as it exists in London. In the first place there is needed at almost every stage provision for individual care and personal influence. Whether in connection with hospital or clinic, poor law infirmary, police-court or prison, personal after-care is of first importance, and before any of these institutions is reached an opportunity for individual help occurs which, wisely used, may prevent untold misery and disease, and much public expenditure. In the year ending August 21, 1920, the Metropolitan Police Women Patrols cautioned 6,676 girls for loitering and accosting men. Of this number 652 girls asked for assistance to escape from their mode of life. Evidence of voluntary workers endorses the great value of such work on the streets, and leads directly to the second consideration which must find a place in any adequate scheme—namely, the provision of suitable and carefully classified institutions for various purposes. Temporary accommodation, always and easily available, is the first need of those whose work lies in the streets. Then there must be a variety of voluntary homes where the initiative and devotion of the individual worker can find full scope, and those under her care learn afresh the lessons of self-discipline and the possibility of self-respect, and so take their place again as good citizens.

Finally, a comprehensive scheme must provide for investigation of the contributory causes of wrong doing and for their removal. Bad housing, unhappy home life, monotony, economic pressure, unsatisfactory conditions of employment, unhealthy amusements or the absence of sufficient scope for wholesome recreation—all these and others are factors in what is, at once, one of the most difficult and most serious of the social problems of a great city. The value of the new Central Council will be measurable, not by any spectacular "results," but by the extent to which it succeeds in broadening the outlook of those who face this problem and in seeing that it is dealt with as a whole. Unrelated, piecemeal efforts will never solve it.

* This figure is quoted from an Appendix to the recent Report by the Medical Officer to the London County Council on the V.D. scheme, in which the social aspects of the V.D. problem are examined at some length.

